# Disclosing azole resistance mechanisms in resistant *Candida glabrata* strains encoding wild-type or gain-of-function *CgPDR1* alleles through comparative genomics and transcriptomics

**DOI:** 10.1093/g3journal/jkac110

**Published:** 2022-05-09

**Authors:** Sara B Salazar, Maria Joana F Pinheiro, Danielle Sotti-Novais, Ana R Soares, Maria M Lopes, Teresa Ferreira, Vitória Rodrigues, Fábio Fernandes, Nuno P Mira

**Affiliations:** iBB, Institute for Bioengineering and Biosciences, Instituto Superior Técnico—Department of Bioengineering, Universidade de Lisboa, Lisboa 1049-001, Portugal; Associate Laboratory i4HB—Institute for Health and Bioeconomy at Instituto Superior Técnico, Universidade de Lisboa, Lisboa 1049-001, Portugal; iBB, Institute for Bioengineering and Biosciences, Instituto Superior Técnico—Department of Bioengineering, Universidade de Lisboa, Lisboa 1049-001, Portugal; Associate Laboratory i4HB—Institute for Health and Bioeconomy at Instituto Superior Técnico, Universidade de Lisboa, Lisboa 1049-001, Portugal; iBB, Institute for Bioengineering and Biosciences, Instituto Superior Técnico—Department of Bioengineering, Universidade de Lisboa, Lisboa 1049-001, Portugal; Associate Laboratory i4HB—Institute for Health and Bioeconomy at Instituto Superior Técnico, Universidade de Lisboa, Lisboa 1049-001, Portugal; Department of Medical Sciences, Institute of Biomedicine (iBiMED), University of Aveiro, Aveiro 3810, Portugal; Departamento de Microbiologia e Imunologia, Faculdade de Farmácia da Universidade de Lisboa, Lisboa 1649-003, Portugal; Laboratório de Microbiologia, Hospital Dona Estefânia (Centro Hospitalar Universitário Lisboa Central), Lisboa 1169-045, Portugal; Seção de Microbiologia, Laboratório SYNLAB—Lisboa, Grupo SYNLAB Portugal, Lisboa 1070-061, Portugal; iBB, Institute for Bioengineering and Biosciences, Instituto Superior Técnico—Department of Bioengineering, Universidade de Lisboa, Lisboa 1049-001, Portugal; Associate Laboratory i4HB—Institute for Health and Bioeconomy at Instituto Superior Técnico, Universidade de Lisboa, Lisboa 1049-001, Portugal; iBB, Institute for Bioengineering and Biosciences, Instituto Superior Técnico—Department of Bioengineering, Universidade de Lisboa, Lisboa 1049-001, Portugal; Associate Laboratory i4HB—Institute for Health and Bioeconomy at Instituto Superior Técnico, Universidade de Lisboa, Lisboa 1049-001, Portugal

**Keywords:** CgPdr1, CgPdr1-dependent and independent azole-resistance, azole resistance, *Candida glabrata*

## Abstract

The pathogenic yeast *Candida glabrata* is intrinsically resilient to azoles and rapidly acquires resistance to these antifungals, in vitro and in vivo. In most cases azole-resistant *C. glabrata* clinical strains encode hyperactive CgPdr1 variants, however, resistant strains encoding wild-type *CgPDR1* alleles have also been isolated, although remaining to be disclosed the underlying resistance mechanism. In this study, we scrutinized the mechanisms underlying resistance to azoles of 8 resistant clinical *C. glabrata* strains, identified along the course of epidemiological surveys undertaken in Portugal. Seven of the strains were found to encode CgPdr1 gain-of-function variants (I392M, E555K, G558C, and I803T) with the substitutions I392M and I803T being herein characterized as hyper-activating mutations for the first time. While cells expressing the wild-type *CgPDR1* allele required the mediator subunit Gal11A to enhance tolerance to fluconazole, this was dispensable for cells expressing the I803T variant indicating that the CgPdr1 interactome is shaped by different gain-of-function substitutions. Genomic and transcriptomic profiling of the sole azole-resistant *C. glabrata* isolate encoding a wild-type *CgPDR1* allele (ISTB218) revealed that under fluconazole stress this strain over-expresses various genes described to provide protection against this antifungal, while also showing reduced expression of genes described to increase sensitivity to these drugs. The overall role in driving the azole-resistance phenotype of the ISTB218 *C. glabrata* isolate played by these changes in the transcriptome and genome of the ISTB218 isolate are discussed shedding light into mechanisms of resistance that go beyond the CgPdr1-signalling pathway and that may alone, or in combination, pave the way for the acquisition of resistance to azoles in vivo.

## Introduction

The increase in the size of the immunocompromised population, resulting from the intensification of medical procedures, has been potentiating the incidence of invasive infections caused by fungi (Pappas *et al.* 2018). Among the more relevant ethiological agents causative of invasive fungal infections are *Candida* species (Bongomin *et al.* 2017; Castanheira *et al.* 2017; Pfaller *et al.* 2019), whose associated infections have high rates of mortality and morbidity (Sanguinetti *et al.* 2015; Strollo *et al.* 2016; Swanson *et al.* 2016; Wan Ismail *et al.* 2020; Zhang *et al.* 2020). Although *Candida*  *albicans* remains the leading infecting *Candida* species, a persistent increase in the incidence of infections caused by non albicans *Candida* species (generally designated as NACS) has been observed worldwide(Pfaller *et al.* 2010, 2019; Sanguinetti *et al.* 2015). This increase is worrisome as infections caused by NACS have higher mortality and morbidity rates than those attributed to *C. albicans* (Horn *et al.* 2009; Sandhu *et al.* 2017; Yeşilkaya *et al.* 2017; Gangneux *et al.* 2018).

Compared to *C. albicans*, NACS are more tolerant to azoles (as reviewed in Salazar *et al.* 2020) and can acquire resistance at a higher rate, specially *Candida*  *glabrata* (Pfaller *et al.* 2019). The increase in azole resistance among *Candida* strains threatens the successful therapeutic utilization of azoles, as confirmed by the prolonged hospital stays and poorer outcomes of patients colonized with azole-resistant strains (Sanguinetti *et al.* 2015). The low susceptibility of *C. glabrata* to azoles is attributed to its capability of bypassing the accumulation of toxic sterols in the plasma membrane caused by the azole-induced inhibition of Erg11, an essential enzyme for ergosterol biosynthesis (Nakayama *et al.* 2001; Hull *et al.* 2012; Salazar *et al.* 2020). While in *C. albicans* azole resistance is largely determined by the occurrence of point modifications in Erg11 coding sequence, in *C. glabrata* this mechanism is very rare (as reviewed in Salazar *et al.* 2020). The formation of mini-chromosomes harboring multiple copies of essential azole-resistance genes or the inactivation of the DNA repair enzyme *CgMSH2* as a mean to increase genetic diversity, are mechanisms described to mediate azole resistance in *C. glabrata* clinical isolates (Polakova *et al.* 2009; Delliere *et al.* 2016; Healey *et al.* 2016; Salazar *et al.* 2020). Although these mechanisms reflect the highly plastic nature of the *C. glabrata* genome (Carreté *et al.* 2018; Carrete *et al.* 2019), which favors the rapid acquisition of resistance to azoles in vivo and in vitro (Carrete *et al.* 2019; Cavalheiro *et al.* 2019), they are not observed to underlie the azole resistance-phenotype of many clinical strains (as reviewed in Salazar *et al.* 2020). In fact, a recent overview of the genes described to influence azole resistance in laboratory strains and those confirmed to underlie azole resistance in clinical strains shows a very modest overlap reflecting the lower amount of work that has been undertaken in clinical strains, compared to laboratory strains (as reviewed in Salazar *et al.* 2020). Also, the occurrence of factors conditioning azole resistance in vivo that may not be mimicked to the studies conducted in vitro should also contribute for some dissimilarities in the findings obtained in laboratory and in clinical strains. In most cases azole resistance in *C. glabrata* clinical strains derives from them acquiring gain-of-function (GOF) mutations in the transcription regulator CgPdr1, a central player in control of response and tolerance to xenobiotics in yeasts (as reviewed in Moye-Rowley 2019). GOF mutations render CgPdr1 constitutively active resulting in a potent up-regulation of target genes even when drugs are not present. The more prominent CgPdr1 target that is up-regulated upon CgPdr1 activation are the drug-efflux pumps CgCdr1 and CgPdh1 (as reviewed in Moye-Rowley 2019; Salazar *et al.* 2020), believed to play an essential role in promoting the active efflux of azoles thereby alleviating the deleterious effects prompted by their internal accumulation. Notably, although the outcome of the occurrence of CgPdr1 GOF mutations appears to be the same (constitutive activation of CgPdr1 and azole resistance) the molecular mechanisms underneath are apparently different since it has been observed that GOFs have a very different impact in *C. glabrata* genomic expression, probably due to different interactions with the transcriptional machinery (Ferrari *et al.* 2011; Salazar *et al.* 2018; Simonicova and Moye-Rowley 2020). CgPdr1 interacts with the complex mediator subunit Gal11A to increase recruitment of transcriptional machinery and promote the up-regulation of target genes (Nishikawa *et al.* 2016). Functional analysis of strains encoding different CgPdr1 GOF alleles have found differences in the way by which these mutants interact with Gal11A with some modifications retaining the need of the wild-type protein for Gal11A, while others appear to be less dependent of it (Simonicova and Moye-Rowley 2020).

Despite the prevalence of CgPdr1 gain-of-function mutants among azole-resistant *C. glabrata* strains, resistant strains encoding wild-type *CgPDR1* alleles have also been isolated (Vermitsky and Edlind 2004; Ferrari *et al.* 2009; Healey *et al.* 2016; Gohar *et al.* 2017; Biswas *et al.* 2018; Hou *et al.* 2018; Arastehfar *et al.* 2019), however, these were not further investigated and therefore the underlying resistance mechanisms remain unknown. In this study, we scrutinized the azole-resistance phenotype of 8 *C. glabrata* resistant strains that we have identified in the course of epidemiological surveys undertaken in Portugal. Seven of these strains were found to encode CgPdr1 variants, with 2 of them, I392M and I803T, being herein characterized for the first time. One strain (ISTB218) was found to encode a wild-type *CgPDR1* allele and was therefore subjected to comparative transcriptomic and genomic analyses (using 2 susceptible strains as references) to shed light into genes and pathways that could mediate azole resistance in vivo, beyond CgPdr1.

## Methods

### Strains and growth media

This work resorted to 1,270 clinical isolates identified as belonging to the *Candida* genus as well as the laboratory strains CBS138, SKY107, and LYS2, as detailed in Supplementary Table 1. Strains were cultivated in YPD, in RPMI or in minimal medium containing glucose or N-acetylglucosamine as the sole carbon sources, being the composition of these media used provided in Supplementary Methods 1.

### Plasmids

Plasmid pSP76, which expresses CgPdr1 from its natural promoter and terminator (Khakhina *et al.* 2018), was used to complement the deletion of *PDR1* in SKY107 background. Using pSP76 as a template, 3 derivative plasmids were generated to allow expression of the *CgPDR1* mutants G822T (yielding the K274Q substitution), T1176G (yielding the I392M substitution), and T2408C (yielding the I803T substitution). Codon substitution was obtained by amplification of the pSP76 plasmid with PfuUltra High-Fidelity DNA polymerase (Agilent) using the mutagenic primers detailed in Supplementary Table 2. The PCR product was treated with 2U DpnI (New England BioLabs) for 1 h at 37°C, followed by self-ligation overnight, at 16°C, using 10U T4 polynucleotide kinase and 400U T4 ligase (both enzymes from New England BioLabs). The resulting product was transformed in *Escherichia*  *coli* DH5α competent cells by classical transformation and constructs verified by Sanger sequencing. To obtain the A12_CgPdr1 plasmid the promotor and sequence of the *CgPDR1* gene were amplified from plasmid pSP76 and were inserted in the NotI/BamHI sits of the pYR29-MycHis plasmid (Merhej *et al*. 2015). Engineering of the mutants A820C (yielding the K274Q substitution), T1176G (yielding the I392M substitution), and T2408C (yielding the I803T substitution) was performed using the same strategy as described above for the constructs performed in the pSP76 backbone.

### Azole susceptibility testing of the isolates

A total of 479 *C. albicans* and *C. glabrata* isolates were profiled for their resistance to fluconazole and voriconazole. In an initial step, the strains were profiled for their growth in the presence of resistance breakpoints concentrations of fluconazole and voriconazole and those exhibiting at least 25% reduction in growth in the presence of the azole, comparing with growth observed in drug-free medium, were selected for a second step. In this second step, the MIC of fluconazole and voriconazole was determined using the EUCAST highly standardized microdilution method (Subcommittee on Antifungal Susceptibility Testing of the EECFAST 2008) (details in Supplementary Information 1).

### Sequencing of *CgPDR1* allele encoded by the azole-resistant *C. glabrata* isolates

To sequence the *CgPDR1* gene encoded by the azole-resistant *C. glabrata* isolates, genomic DNA was obtained and used as a template to amplify, by PCR, the *CgPDR1* gene (details in Supplementary Information 1). The PCR product was sequenced (at least twice and using independent PCR products) at STABVIDA as a service.

### Assessment of *CgCDR1* and *CgPUP1* expression in azole-resistant strains

The transcript levels of *CgCDR1* and *CgPUP1* genes were compared in the 7 azole-resistant strains and in CBS138 during exponential growth in RPMI medium (details on Supplementary Information 1). Conversion of the recovered RNA from the different cultures into cDNA was performed using 1 μg of RNA in the C1000 Thermal Cycler (Bio-Rad, Hercules, USA). The subsequent quantitative PCR step was performed using 2.5 μl of the cDNA and SYBR Green super mix (BioRad) in the 7,500 Real Time PCR System (Applied Biosystems). Primer sequences used are available in Supplementary Table 2. Gene expression was calculated using gene *RDN25* as an internal control.

### Transcriptomic and genomic profiling of isolates FFUL443, FFUL674, ISTB218, and ISTA29

Transcriptomic profile of isolates FFUL443, FFUL674, and ISTB2018 was compared with the one of the laboratory strain CBS138 during exponential growth in RPMI medium, either or not supplemented with 32 mg/L of fluconazole, using a species-specific DNA microarray for *C. glabrata* (Rossignol *et al.* 2007) (details on Supplementary Information 1). Whole-genome sequencing of ISTB218 (azole resistant) and ISTA29 (azole susceptible) was performed at CD Genomics Inc (USA), as a paid-service, and the reads obtained were used for SNP calling using the tools available at CLC Genomics Workbench software (further details in Supplementary Information 1).

**Fig. 1.**
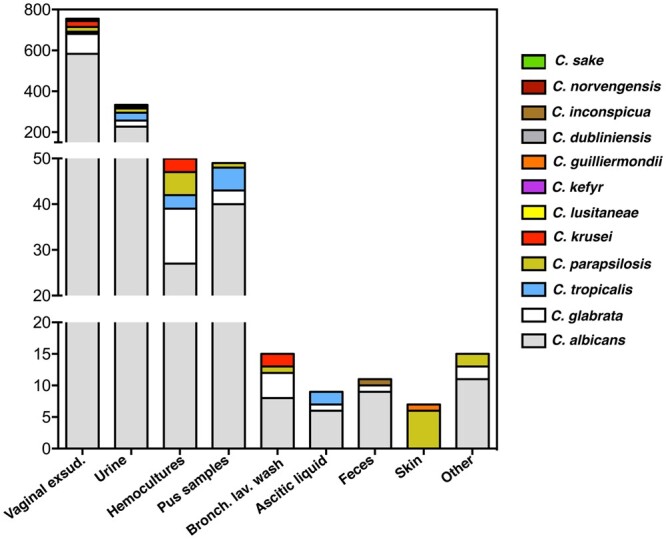
Species-distribution of the collection of *Candida* isolates (identified as belonging to a species of the Candida genus based on MALDI-TOF profiling) examined in this work according with the product they were retrieved from.

*Susceptibility assays to fluconazole of* Δ*pdr1* and Δ*pdr1*Δ*Gal11A C. glabrata cells expressing different CgPDR1 alleles.* SKY107 (Δ*pdr1*) cells or LYS2 (Δ*pdr1*Δ*Gal11A*) cells were transformed with the pSP76 or A12_CgPDR1 plasmids to drive expression of the wild-type *CgPDR1* allele or with the engineered plasmids to drive the expression of the GOF alleles K274Q, I392M, or I803T. The transformants were cultivated overnight, at 30°C and with an orbital agitation of 250 rpm, in minimal medium without uracil and on the next day the cells were re-inoculated in fresh MM medium at an OD_600nm_ of 0.1. The cells were left to grow (at 30°C and with an orbital agitation of 250 rpm) until mid-exponential phase (DO_600nm_∼0.8) and used to prepare a cell suspension (in water) with an OD_600nm_ of 0.05. Four microliters of this cell suspension and of corresponding 1:4 (lane b shown in Fig. 3) and 1:16 (lane c shown in Fig. 3) of it were applied as spots onto the surface of solid MM or YPD either or not supplemented with the indicated concentrations of fluconazole. For the determination of the MICs in these transformants the same procedure as detailed above for the clinical isolates was used.

## Results

### Distribution of *Candida* species among a cohort of isolates recovered across epidemiological surveys undertaken in Portugal

For the present study, we made use of 1,270 *Candida* clinical isolates collected from patients attending hospitals in the Lisbon area, in Portugal, between 2015 and 2017. *Candida*  *albicans* was, by far, the species more frequently isolated comprising 922 isolates, followed by *C. glabrata* (154 isolates), *Candida*  *tropicalis* (62 isolates), *Candida*  *parapsilosis* (61 isolates), *Candida*  *krusei* (40 isolates), *Candida*  *lusitaniae* (12 isolates), *Candida*  *kefyr* (10 isolates) and the rare *Candida*  *guilliermondii* (2 isolates), *Candida*  *dubliniensis*, *Candida*  *sake*, and *Candida*  *inconspicua* (1 isolate each) (Fig. 1 and Supplementary Table 1). Around 92% of the isolates examined were retrieved from nonsterile sites including vaginal exudates, urine, skin or feces, the remaining being retrieved from sterile products like hemocultures (Supplementary Table 1). *Candida*  *albicans* was the more frequent species isolated from all types of products, in line with the described versatility of this species as a human colonizer (Romo and Kumamoto 2020). *Candida*  *glabrata* was, in almost all cases, the second more frequent species isolated (Fig. 1). It was of note the isolation of *C. tropicalis*, *C. parapsilosis*, *C. krusei*, and *C. kefyr* in hemocultures, consistent with their reported ability to cause invasive candidiasis, including *C. kefyr* whose relevance in candidemia is poorly studied but increasing (Dufresne *et al.* 2014; Khan *et al.* 2015). Since MALDI-TOF cannot clearly distinguish *C. albicans* from its closely related variant *C. albicans var*  *africana* (Tietz *et al.* 1995; Chowdhary *et al.* 2017) and in Portugal this biovariant has not been described, we profiled our cohort of *C. albicans* strains for growth in minimal media having N-acetylglucosamine as the sole source of carbon as *C. albicans* var *africana* cells are unable to use this carbon source(Tietz *et al.* 1995). Eight *C. albicans var africana* strains could be identified, 7 being recovered from vaginal exudates, consistent with the human genitourinary tract being its primary colonization niche (Tietz *et al.* 1995; Chowdhary *et al.* 2017; Supplementary Table 1).

### Profile of resistance to fluconazole and voriconazole among the *C. albicans* and *C. glabrata* strains

We randomly selected 401 *C. albicans* and 78 *C. glabrata* strains (details in Supplementary Table 1) for a profile of resistance to fluconazole and voriconazole. The strains were first phenotyped for their ability to grow in the presence of concentrations of fluconazole and voriconazole equal to the resistance breakpoints defined by EUCAST (32 mg/L of fluconazole and 1 mg/L of voriconazole for *C. glabrata* and 4 mg/L of fluconazole and 0.25 mg/L of voriconazole for *C. albicans*). Twenty-six isolates exhibiting a growth reduction in the presence of the azoles of, at least, 25% the growth registered in drug-free medium were selected for individual determination of the MIC of the 2 azoles (Supplementary Table 1). This analysis led to the identification of 13 *C. albicans* and 4 *C. glabrata* isolates with MICs for fluconazole and voriconazole above the resistance breakpoint, while 2 *C. albicans* isolates (ISTB16 and ISTB284) could only be considered resistant to fluconazole (further details in Supplementary Table 1).

### Sequencing of the *CgPDR1* allele encoded by the azole-resistant *C. glabrata* isolates

We focused then on the 4 identified *C. glabrata* azole-resistant strains identified in our cohort: ISTB218, ISTA56, ISTB607, and ISTB556. We started by examining the sequence of the *CgPDR1* allele encoded by these strains with the results being shown in Table 1 (Supplementary Table 3 provides further details). We also included in this analysis 3 *C. glabrata* azole-resistant strains (FFUL443, FFUL674, and FFUL830) recovered from previous epidemiological surveys and that were also identified by our laboratory as azole-resistant but whose underlying resistance mechanism was not further characterized (Salazar *et al.* 2018). The *CgPDR1* gene encoded by all the examined strains exhibited the nonsynonymous substitutions S76P, V91I, L98S, T143P, and/or D243N, previously described to occur both in azole susceptible and resistant *C. glabrata* strains (Ferrari *et al.* 2009; Biswas *et al.* 2018; Hou *et al.* 2018; Carrete *et al.* 2019; Tantivitayakul *et al.* 2019) (Table 1 and Supplementary Table 3). Two of the azole-resistant isolates encode the demonstrated R376W (ISTB556, ISTB607) GOF CgPdr1 variant (Ferrari *et al.* 2009; Katiyar *et al.* 2016), while isolates FFUL830, ISTA56, and FFUL674 encode *CgPDR1* alleles with the E555K, G558C, and I803T substitutions. All these substitutions were previously identified in azole-resistant strains but not in susceptible ones for which they were considered to represent CgPdr1 GOF variants and to mediate the azole-resistance phenotype of the strains in which they were identified (Ferrari *et al.* (2009); Katiyar *et al.* (2016). In line with this idea, isolates expressing these *CgPDR1* alleles (IST866, ISTA56) over-express (comparing to the susceptible reference CBS138 strain) the CgPdr1-targets *CgCDR1* and *CgPUP1* during growth in unsupplemented RPMI medium, a phenotype observed in strais expressing *CgPDR1* GOF alleles (Ferrari *et al.* 2009) (Supplementary Figure S1). Isolate FFUL443 was found to encode a CgPdr1 allele with a not previously described substitution, I392M, while ISTB218 strain encoded a *CgPDR1* allele with no other modification besides those also observed in the susceptible strains (Table 1).

**Table 1.** Results obtained upon sequencing of the *CgPDR1* gene from the seven azole-resistant *C. glabrata* strains examined in this study.

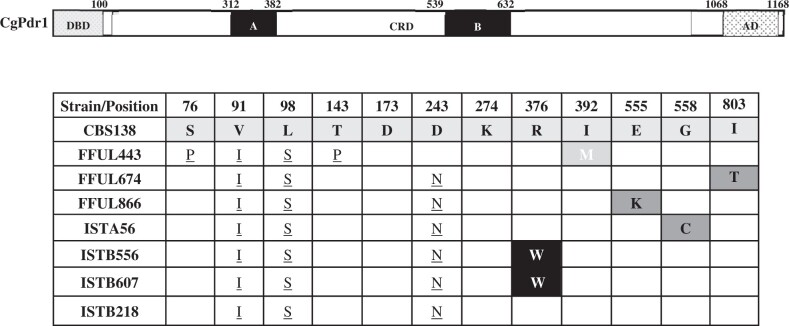

The non-synonymous modifications found in the coding sequencing of the *CgPDR1* gene encoded by the strains is compared with the one of the azole-susceptible reference strain CBS138. Those modifications demonstrated before to serve as CgPdr1 GOF variants are marked in dark grey boxes, while those previously reported in azole-resistant strains but not in susceptible ones are indicated in black boxes. SNPs described in azole susceptible and resistant strains are underlined. The herein described I392M substitution is indicated in the light grey box. The functional domains of CgPdr1 are also indicated in the schematic representation provided that shows the different domains mapped in the regulator: DBD, DNA binding domain; CRD, Central Regulatory Domain (in black are highlighted the two regions where most GOFs have been identified; TAD, transactivation domain).

**Table 2.** Results from comparative transcriptomic analysis between the azole-resistant *C. glabrata* strain ISTB218 and the azole-susceptible strain CBS138 during growth in RPMI medium supplemented with 32 mg/L fluconazole.

ORF name	Gene name	*Sc orth.*	mRNA ISTB218/mRNA CBS138	Function
***Metabolism and transport of sterols***				
*CAGL0F01419g*	*CgAUS1*	*AUS1*	11.6	ATP-binding cassette transporter involved in sterol uptake
*CAGL0F05137g*		*PRY2*	7.3	Ortholog(s) have role in protein lipoylation
*CAGL0F07865g*	*CgUPC2B*	*UPC2*	4.8	Transcriptional regulator of ergosterol biosynthesis
*CAGL0D05434g*	*CgROX1*	*ROX1*	2.6	Protein involved in regulation of ergosterol biosynthesis
*CAGL0L10142g*	*CgRSB1*	*RSB1*	2.2	Putative sphingolipid flippase
*CAGL0L10714g*	*CgERG2*	*ERG2*	−2.8	C-8 sterol isomerase
*CAGL0K01353g*		*NPC2*	−2.9	Ortholog(s) have sterol binding activity, role in intracellular sterol transport
*CAGL0E06424g*				Ortholog(s) have cytochrome-b5 reductase activity; role in ergosterol biosynthetic process
*CAGL0L08888g*		*NCR1*	−3.3	Ortholog(s) have sterol binding activity, role in sphingolipid metabolic process, sterol transport
***Transport of xenobiotics***				
CAGL0G08624g	* CgQDR2*	*QDR2*	2.7	Multidrug resistance transporter of the MFS Superfamily
*CAGL0J01661g*			2.3	Predicted role in transmembrane transport
*CAGL0A01221g*		*AQY1*	−8.7	Predicted acquaporine
*CAGL0D00154g*		*AQY1*	−7.5	Predicted acquaporine
*CAGL0F02717g*	*CgPDH1*	*PDR15*	2.1	Multidrug resistance transporter of the ABC Superfamily
CAGL0G03927g	*CgTPO1_1*	*TPO1*	2.0	Multidrug resistance transporter of the MFS Superfamily
CAGL0H00847g		*HUT1*	−2.8	Ortholog(s) have UDP-galactose transmembrane transporter activity
***Transcription***				
CAGL0L03377g	*CgZCF31*	*SIP34*	3.8	Putative transcription factor
CAGL0L07480g		*NRG2*	3.1	Predicted transcription factor
***Adhesion***				
*CAGL0G10175g*	*CgAWP6*	*DAN1*	52.3	Predicted adhesin
*CAGL0H07469g*		*ICS2*	14.8	Predicted adhesin
CAGL0M14069g	*CgPWP6*	*FLO9*	11.4	Adhesin-like protein
CAGL0E06644g	*CgEPA1*	*FLO1*	−2.5	Subtelomerically encoded adhesin with a role in cell adhesion
CAGL0C00110g	*CgEPA6*	*FLO1*	−9.9	Subtelomerically encoded adhesin with a role in cell adhesion
CAGL0E06666g	*CgEPA2*	*FLO1*	−7.6	Subtelomerically encoded adhesin with a role in cell adhesion
CAGL0K13024g	*CgEAD1*	*-*	−5.8	Adhesin-like protein required for adherence to endothelial cells
CAGL0K00110g	*CgAWP2*	*-*	−5.7	Putative adhesin
***Other functions***				
CAGL0I07843g	* CgADH1*	*ADH1*	6.5	Putative alcohol dehydrogenase isoenzyme II
CAGL0K01991g		*NCL1*	2.6	Predicted role in tRNA methylation
CAGL0L12320g		*GEP5*	−2.3	Role in mitochondrial genome maintenance
*CAGL0K05797g*	*CgEMI1*	*EMI1*	−3.8	Ortholog(s) have role in mitochondrion organization
*CAGL0K01419g*		*MTF2*	−3.7	Ortholog(s) have RNA binding activity, role in mRNA processing
CAGL0K00715g		*RTA1*	−6.4	Predicted lipid-translocating exporter; over-expression of ortholog confers tolerance to the ergosterol biosynthesis inhibitor 7-aminocholesterol
*CAGL0F00957g*		*TPD3*	−3.3	Putative serine/threonine protein phosphatase 2A
CAGL0A02299g		*-*	−12.1	Unknown function

A selected set of genes found to be differently transcribed (above or below 2-fold) in the 2 strains is shown in this table, along with the description of their function and the corresponding ortholog in *S. cerevisiae*. Genes with a protective function against fluconazole are highlighted in light gray, while those whose deletion was found to result in enhanced tolerance to azole are highlighted in dark gray. The protective effect against fluconazole of the genes was based on results from Nagi *et al.* (2011); Gupta *et al.* (2017); Gale *et al.* (2020).

### The I803T and I392M substitutions are new GOF *CgPdr1* variants, as unveiled by comparative transcriptomics and detailed molecular analyses

**Fig. 2.**
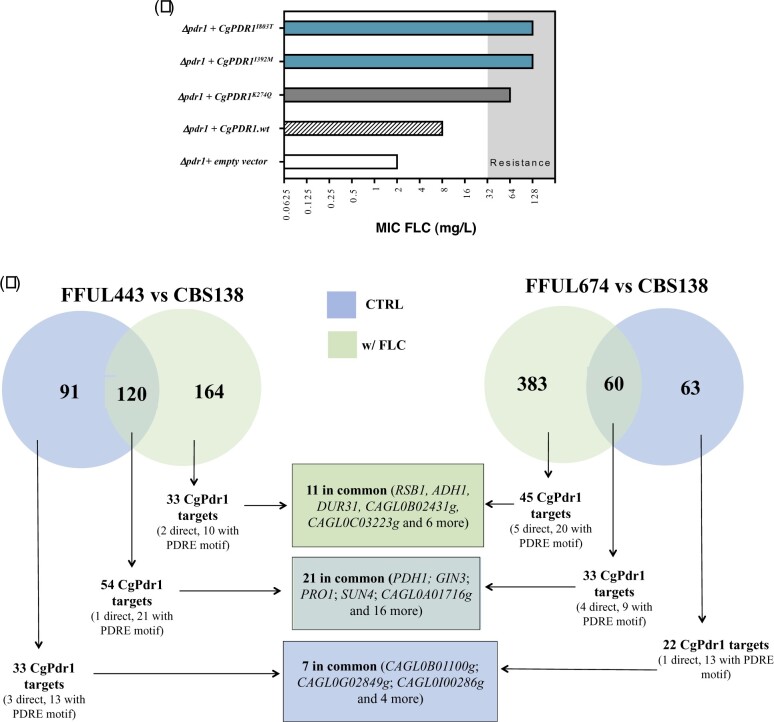
Effect in tolerance to fluconazole and in genomic expression of *C. glabrata* of the expression of the CgPdr1^I392M^ or CgPdr1^I803T^ variants (a) MIC for fluconazole obtained for SKY107 cells transformed with plasmid pSP76 which drives expression of *CgPDR1* from its natural promoter and terminator and in the derived plasmids that encode the *CgPDR1* allele with the individual substitutions I392M and I803T. As a control, the described CgPdr1 GOF variant K274Q was also used. In gray it is highlighted the MIC values above the defined resistance breakpoint for fluconazole; b) Comparative genomic expression between the azole-susceptible strain *C. glabrata* CBS138 and the azole-resistant isolates FFUL443 (clinical strain that harbors the I392M modification) and FFUL674 (clinical strain that harbors the I803T modification) during growth in RPMI medium (in blue) or in this same medium supplemented with fluconazole (32 mg/L; in green). Genes found to be over- or under-expressed in the clinical strains (above a 2-fold threshold level) were selected for this comparative analysis and among these documented as being positively regulated by CgPdr1 identified and distinguished between direct and indirect targets, based on the information available in the PathoYeastract database (Monteiro *et al.* 2017). The data that supported design of the data presented in this figure is detailed in Supplementary Tables 4–7.

The azole-resistant *C. glabrata* isolate FFUL443 encodes a *CgPDR1* allele with a not previously described substitution, I392M, while the FFUL674 isolate encodes a variant, I803T, that was observed in an azole-resistant strain (Katiyar *et al.* 2016) but not further studied. We hypothesized that these substitutions could represent new CgPdr1 gain-of-function substitutions and to clarify this aspect we ectopically expressed the wild-type *CgPDR1* allele, as well as the *CgPDR1^I803T^* and *CgPDR1^I392M^* variants, in a Δ*pdr1* background. Consistent with the idea that I392M and I803T substitutions are gain-of-function mutations, Δ*pdr1* cells expressing the I803T and I392M variants are more tolerant to fluconazole than those expressing the wild-type version of CgPdr1 (Fig. 2a). Global comparative transcriptomic analysis of strains FFUL443 and FFUL674 (that express, respectively, the I392M and I803T variants) with the azole-susceptible laboratory strain CBS138 (which expresses the wild-type CgPdr1) in drug-free RPMI medium revealed an over-expression in the clinical strains of 87 and 44 genes described to be activated by CgPdr1 (Supplementary Tables 4 and 6; and results compiled in Fig. 2b) confirming that these strains encode a hyper-active *CgPDR1* allele that bypasses azole-induced activation. Among the up-regulated genes in the clinical strains were *CgCDR1* and *CgPUP1*, the sole 2 CgPdr1 target genes found to be commonly up-regulated in strains encoding different CgPdr1 GOF mutants (Ferrari *et al.* 2009, 2011) (up-regulation of these 2 genes detected in the microarray experiments was further confirmed by real time RT-PCR, Supplementary Fig. 1). Exposure to fluconazole (32 mg/L) caused, as expected, dramatic modifications in the transcriptome of all strains resulting in a higher number of differentially expressed genes (see Venn diagram shown in Fig. 2b and more specific data on the individual gene expression in the presence or absence of fluconazole in Supplementary Tables 4–7). Among the many genes found to be differently expressed between the clinical isolates and the laboratory CBS138 strain, fluconazole-stressed FFUL443 cells over-express 87 documented CgPdr1 targets, 54 of these already being over-expressed in the absence of the azole (Fig. 2b). Azole-challenged FFUL674 cells over-expressed 77 documented CgPdr1-targets, 33 of these already being over-expressed in control conditions (Fig. 2b). Notably, the set of CgPdr1-target genes commonly up-regulated in FFUL443 and FFUL674 cells, either in the presence or absence of fluconazole, was very limited (see Venn diagram in Fig. 2b) confirming previous results sustaining that different gain-of-function substitutions exert a differential impact in *C. glabrata* genomic expression (Ferrari *et al.* 2009; Salazar *et al.* 2018).

### The mediator subunit Gal11A exerts a differential role in conferring tolerance to fluconazole in *C. glabrata* cells expressing different CgPdr1 GOF variants

**Fig. 3.**
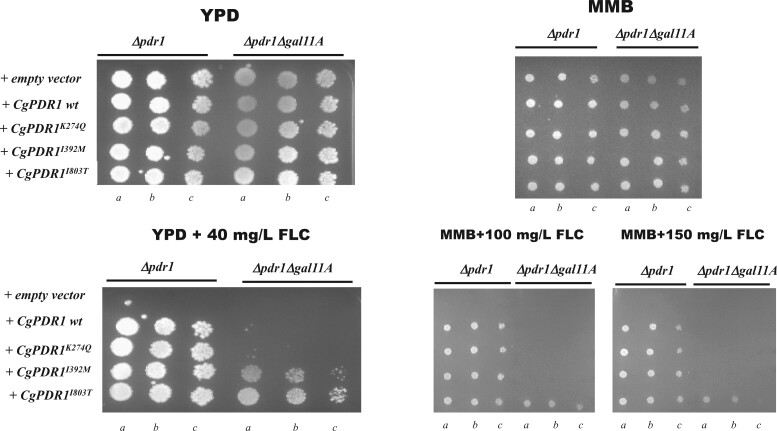
Influence of the Gal11A mediator subunit in tolerance to fluconazole of *C. glabrata* cells expressing wild-type or the GOF CgPdr1 variants K274Q, I392M, and I803T. Δ*pdr1* or Δ*pdr1*Δ*Gal11A* cells were transformed with the A12_*CgPDR1* plasmid (which drives expression of *CgPDR1* from its natural terminator and promoter) or with the derived plasmids A12_*CgPDR1*^K274Q^, A12_*CgPDR1*^I803T^, or A12_*CgPDR1*^I392M^ which drive expression of the corresponding GOF variants and used to compare susceptibility to fluconazole in minimal medium (MM) or in YPD rich medium, as detailed in materials and methods. Cellular suspensions dropped in lanes b) and c) are, respectively, 1:4 and 1:16 dilutions of the cellular suspension dropped in lane a).

The role of the mediator complex in activating CgPdr1 upon exposure of *C. glabrata* cells to azoles has been demonstrated (Thakur *et al.* 2008) although different GOF variants have been found to depend on different degrees of this transcriptional regulatory complex (Simonicova and Moye-Rowley 2020). In this context, we examined the effect of deleting Gal11A, the mediator subunit described to interact with Pdr1 (Thakur *et al.* 2008), in the ability of the *CgPDR1* I392M and I803T alleles to confer tolerance to fluconazole in *C. glabrata* (Fig. 3). For the sake of comparison, we have also included in this experiment the previously characterized *CgPDR1* K274Q GOF allele (Salazar *et al.* 2018). As expected, the ability of a wild-type *CgPDR1* allele to restore tolerance to fluconazole in a Δ*pdr1* background was fully dependent of *GAL11A* (Fig. 3). Δ*pdr1* cells expressing the CgPdr1^K274Q^ variant also exhibited some dependence of the Gal11A subunit, although lower than the one exhibited by the wild-type CgPdr1 variant (Fig. 3). Differently, Δ*pdr1* cells expressing CgPdr1^I803T^ bypassed the requirement for Gal11A to confer protection against fluconazole, a phenotype that was also obtained upon the expression of the CgPdr1^I392M^ variant, albeit at a lower extent (Fig. 3). A similar trend of results was obtained upon determination of the MICs for fluconazole for Δ*pdr1* and Δ*pdr1*Δ*gal11A* cells expressing the different CgPdr1 variants (Supplementary Fig. 2). On the overall the herein obtained results demonstrate that cells expressing the CgPdr1 I803T and, less significantly, I392M and K274Q variants show a considerable lower dependence for Gal11A in improving tolerance to fluconazole than cells expressing the wild-type version of CgPdr1, consistent with the previously reported idea that different modifications in the coding sequence of CgPdr1 may result in a stronger or weaker need for the mediator complex to promote fluconazole tolerance (Simonicova and Moye-Rowley 2020).

### OMICS profiling of the azole-resistant isolate *C. glabrata* ISTB218, encoding a “wild-type” *CgPDR1* allele

The azole-resistant isolate *C. glabrata* ISTB218 that encodes a *CgPDR1* allele not having nonsynonymous substitutions that could be linked with azole-resistance was subjected to a genomic and transcriptomic profiling (in the presence or absence of fluconazole) to elucidate the underlying resistance mechanism. During cultivation in RPMI growth medium 490 genes could be considered differently expressed (above a threshold level of 2-fold) between the ISTB218 isolate and the azole-susceptible strain CBS138, 245 of these being more transcribed in the isolate and 252 genes more expressed in the laboratory strain (Supplementary Table 8, Supplementary Fig. 3). Within the dataset of genes over-expressed in ISTB218, we could find only 1 gene documented to be directly regulated by CgPdr1 (CAGL0A01650g), consistent with this strain encoding a “wild-type” CgPdr1, inactive when cells are growing in the absence of a xenobiotic (Moye-Rowley 2019). Exposure to fluconazole caused profound changes in genomic expression of ISTB218 and CBS138 cells resulting in an increased number of differently expressed genes (as detailed in Supplementary Fig. 3 and Supplementary Tables 8 and 9). In particular, we could detect 302 genes over-expressed in fluconazole-stressed ISTB218 cells (203 already found to be up-regulated in the absence of the azole) while 250 genes were more expressed in CBS138 (143 already being up-regulated in this strains in the absence of fluconazole). The higher tolerance to fluconazole of ISTB218 cells could result from them expressing at higher levels genes documented to confer protection against this antifungal. Therefore we systematically searched our list of up-regulated genes in ISTB218 for described azole-resistance genes and identified 10 azole-resistance genes that are identified in Table 2. Among these genes was *CgAUS1*, encoding an ABC transporter involved in the uptake of exogenous sterols; the alcohol dehydrogenase *CgADH1*; *CgUPC2B*, a transcription factor found to be involved in regulation of ergosterol biosynthesis and implicated in tolerance to fluconazole under hypoxic conditions (Gupta *et al.* 2017); and the multidrug resistance transporters *CgPDH1*, *CgQDR2*, and *CgTPO1_1*. Notably, the gene found to be more significantly down-regulated in fluconazole-challenged ISTB218 cells, *CAGL0A02299g*, encoding a protein of unknown function, was described as an azole-susceptibility gene (that is, a gene whose deletion enhances tolerance to fluconazole) (Gale *et al.* 2020). Similarly, 5 other azole-susceptibility genes were also found to be down-regulated in the ISTB218 strain during fluconazole challenge: the phosphatase *TPD3*; *EMI1*, and *CAGL0L12320g*, required for mitochondrial genome organization and *CAGL0H00847g*, *CAGL0K01419g*, and *CAGL0A02299g*, encoding proteins with uncharacterized functions (as detailed in Table 2). A noticeable result that emerged from close inspection of both the dataset of up- and down-regulated genes in ISTB218 was the dramatic alteration registered in the expression of a large number of adhesin-encoding genes, specially under fluconazole stress (for example, *AWP6* was up-regulated more than 50-fold in fluconazole-stressed ISTB218 cells while in control conditions the difference in the transcript level of this gene was not above the 2-threshold level used) (see details in Table 2 and in Supplementary Tables 8 and 9).

**Table 3.** Single nucleotide polymorphisms (SNPs) were identified in the gene sequences encoded by the azole-resistant ISTB218 strain but not by the azole susceptible strains ISTA29 and CBS138, as suggested by comparative genomic analyses.

ORF name	Gene name	*Sc* ortholog	Function	Nonsynonymous SNPs in the ISTB218 allele (comparing with CBS138)
***Metabolism and transport of sterols***				
*CAGL0F03267g*		*LAM4*	Orthologs bind and perform intermembrane transfer of sterols	Insertion of LysSerAspAlaHisSer between Ser231 and His232
*CAGL0C03872g*	*CgTIR3*	*TIR3*	Putative GPI-linked cell wall protein involved in sterol uptake	Deletion between Val152 and Ser166
***Cell signaling***				
CAGL0J01892g	*CgPAN1*	*PAN1*	Ortholog(s) have role in actin cortical patch assembly,	Gln104fs
*CAGL0I06512g*	*CgBEM2*	*BEM2*	Ortholog(s) have a role in actin cytoskeleton organization	Val1095Ile
*CAGL0B02211g*	*CgCCH1*	*CCH1*	Putative calcium transporter; required for viability upon prolonged fluconazole stress	Tyr1585Ser
CAGL0J09702g	*CgACK1*	*ACK1*	Ortholog(s) have role in regulation of the cell wall integrity pathway	Gly627fs
CAGL0J00539g	*CgSLT2*	*SLT2*	Protein kinase mediating the cell wall integrity pathway	Lys275Gln
***Transcription***				
CAGL0K01727g	*CgRPN4*	*RPN4*	Tanscription factor required for regulation of proteasome-encoding genes	Insertion of AlaGln between Gln99 and Met100
CAGL0L00583g		*USV1*	Ortholog(s) have role in carbon catabolite activation of transcription from RNA polymerase II promoter	Lys175fs
CAGL0J07370g	*CgJJ1*		Negative regulator of fluconazole resistance; mutation causes elevated expression of multidrug transporters *CDR1* and *PDR1*	Ala270Thr
***Transport***				
CAGL0I04862g	*CgSNQ2*	*SNQ2*	Plasma membrane ABC transporter	Pro1104His
CAGL0M07293g		*PDR12*	Plasma membrane ABC transporter	Tyr25His
CAGL0D00154g		*AQY1*	Has domain(s) with predicted channel activity	Phe49fs
***Mitochondrial function***				
*CAGL0J00847g*	*CgSDH1*	*YJR045w*	Ortholog(s) have succinate dehydrogenase activity	Ala41fs

It is indicated the alteration found in the coding sequence of the gene encoded by the ISTB218 strain as well as the biological function attributed to this gene and the corresponding ortholog in *S. cerevisiae*. Genes with a protective function against fluconazole are highlighted in light gray, while those whose deletion was found to result in enhanced tolerance to azole are highlighted in dark gray. The protective effect against fluconazole of the genes was based on results from Gale *et al.* (2020)  *f.s.* denotes frame-shifts resulting in truncation.

**Fig. 4.**
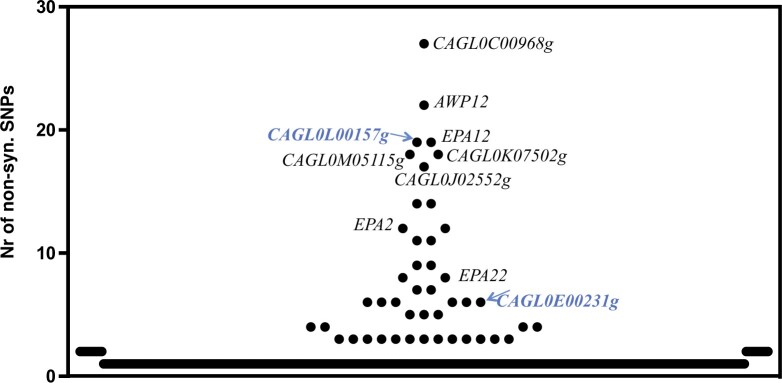
Number of nonsynonymous single-nucleotide polymorphisms (SNPs) found upon comparison of gene sequences encoded by the azole-resistant strain ISTB218 and the azole-susceptible strains CBS138 and ISTA29. The names of adhesin-encoding genes are depicted in the figure to denote the high number of SNPs found in these sequences encoded by the azole-resistant strain ISTB218. Those adhesins that are described to provide protection against azoles in *C. glabrata* are highlighted in blue.

Considering that prior genomic comparative analyses between *C. glabrata* clinical isolates and the CBS138 strain revealed massive number of SNPs that rendered difficult the establishment of relevant genotype to phenotype associations (Vale-Silva *et al.* 2017; Salazar *et al.* 2018; Carrete *et al.* 2019), in this work, we have not only obtained the whole-genomic sequence of ISTB218 but also of an azole-susceptible clinical strain recovered during our phenotypic survey (ISTA29). The data obtained from this strain was used to further “filter” the differences found between ISTB218 and CBS138. Using this approach, we could identify 700 nonsynonymous SNPs that were only found in the azole-resistant strain ISTB218 but not in the azole-susceptible strains CBS138 and ISTA29 (detailed in Supplementary Table 10). We could not detect SNPs in the coding sequences of CgMsh2 or CgErg11, in line with previous genomic analyses undertaken with other azole-resistant isolates that also failed to identify modifications in these genes (Vale-Silva *et al.* 2017; Salazar *et al.* 2018). It was evident that the genes involved in adhesion were those more clearly divergent between ISTB218, ISTA29, and CBS138 strains (Fig. 4), this being in line with the results obtained in previous genomic comparisons involving CBS138 and clinical strains (Vale-Silva *et al.* 2017; Salazar *et al.* 2018). Seventeen documented azole-resistance genes were found to harbor specific SNPs in the azole-resistant ISTB218 strain including the complex mediator sub-unit Gal11B, the adhesins CAGL0L00157g and CAGL0C00231g, the protein kinase Slt2, the ABC transporters CgSnq2 and CgPdr12, the transcriptional regulator CgRpn4 or the sterol transporters CgTir3 and *CAGL0F03267g* (as detailed in Table 3 and in Supplementary Table 10). We could also identify 20 genes exhibiting frame-shifts leading to premature truncations in ISTB218 isolate (as detailed in Table 3 and in Supplementary Table 10). Of these, only the inactivation of *CAGL0J00847g*, a subunit of the succinate dehydrogenase complex, was described to result in improved tolerance to azoles (as detailed in Table 3 and in Supplementary Table 10). The observed truncation in the ISTB218 strain of the aquoporine *CAGL0D00154g*, also observed in another azole-resistant clinical strain (Vale-Silva *et al.* 2017), was interesting considering that this gene was also strongly down-regulated (about 6-fold) in ISTB218 cells exposed to fluconazole. Notably, under fluconazole stress the transcript levels of *CAGL0A01221g*, also predicted to encode an aquoporine, were also much lower (around 8-fold) in ISTB218 than in CBS138 (as detailed in Table 2 and in Supplementary Table 9).

## Discussion

In this study, we shed light into the mechanisms of resistance to azoles in *C. glabrata* clinical strains, an essential knowledge to understand how this pathogenic species acquires resistance in vivo, specially considering that many observations concerning azole resistance in laboratory strains often differ in clinical strains (e.g. reviewed in Sanguinetti *et al.* 2015; Salazar *et al.* 2020). Azole resistance of 6 of the 7 *C. glabrata* resistant isolates that we characterized in this work was linked with the expression of CgPdr1 gain-of-function variants including the already described R76W, the suggested G558C, E555K, and I803T; and I392M, described here for the first time. Like many gain-of-function mutations described in CgPdr1, I392M, G558C, E555K, and I803T substitutions map in the central regulatory domain (CRD) of CgPdr1, with I803T being already close to the activation domain—located approximately in the 50 C-terminal amino acids (Moye-Rowley 2019). While mutations embedded in the central regions of the CRD are believed to relief its inhibitory effect over the transactivation domain, the effect of those located more closely to the activation domain is less clear, as recently shown (Moye-Rowley 2019; Simonicova and Moye-Rowley 2020). Comparative analysis of the transcriptomes of FFUL674 and FFUL443 isolates, encoding, respectively, the I803T and I392M *CgPDR1* alleles, reveals modest overlaps, both in the presence or absence of fluconazole (Fig. 3). This reflects well the differential effect displayed by different GOF mutations over *C. glabrata* genomic expression, as observed before (Vermitsky and Edlind 2004; Salazar *et al.* 2018). Among the set of documented CgPdr1 targets over-expressed in the clinical strains there is a low percentage of direct targets (that is those in which CgPdr1 was found bound in vivo), as detailed in Fig. 3 and in Supplementary Tables 4–9. Consistently, the PDRE motif (the binding site for CgPdr1) was absent from the majority of these documented CgPdr1 targets over-expressed in the clinical strains (detailed in Fig. 3 and in Supplementary Tables 4–9). Altogether these observations suggest that the effect of the GOF mutations in the CgPdr1-mediated alterations of the transcriptome of the clinical strains is mostly indirect. In this context it is particularly interesting the herein reported observation that cells expressing the CgPdr1 I803T, and less significantly the I392M and K274Q alleles, have little dependence of the mediator complex subunit Gal11A to induce azole tolerance, contrasting with the strong dependence exhibited by cells expressing the wild-type allele. These observations support the idea that GOFs modify the CgPdr1 interactome and this is a topic that has to be addressed in further studies to understand how this happens at the biochemical level and what is the impact in the modification of these CgPdr1-interactors in the overall regulation of *C. glabrata* genomic expression, specially under fluconazole stress.

The sole azole-resistant *C. glabrata* isolate identified in our study encoding a *CgPDR1* allele not having substitutions linked with azole resistance was subjected to comparative transcriptomic and genomic analyses with 2 azole susceptible strains, this being the first exhaustive analysis of a strain with these characteristics. The fact that in a randomly selected cohort of 7 azole-resistant strains only one does not encode a gain-of-function CgPdr1 allele demonstrates the preponderance of this mechanism in driving resistance in *C. glabrata*, as also observed in in vitro evolution studies (Cavalheiro *et al.* 2019). Transcriptomic analysis of fluconazole-stressed ISTB218 cells revealed that these cells over-express (comparing with the transcript levels produced in CBS138 cells challenged with the same concentration of fluconazole) several genes described to confer protection against azoles out of which those encoding the drug-efflux pumps *CgPDH1*, *CgQDR2*, and *CgTPO1-1* and the sterol importer *CgAUS1*, up-regulated by more than 10-fold, stood out. *CgPDH1* and *CgQDR2* are known targets of the CgPdr1-regulatory network and therefore their higher level of expression in azole-stressed ISTB218 cells was surprising considering that these encode, like the CBS138 strain, a wild-type *CgPDR1* allele and thus the activation of the regulator induced by fluconazole was expected to be the same for the 2 strains. *CgAUS1* is under the regulation of the ergosterol biosynthesis regulators CgUpc2A and CgUpc2B (Nagi *et al.* 2011). Interestingly, *CgUPC2B* was over-expressed in fluconazole-stressed ISTB218 cells and although this gene plays a negligible role in tolerance to azoles in normoxia conditions, compared to its homolog CgUpc2A, under microaerophilic conditions the expression of *CgUPC2B* was detrimental for protection against fluconazole (Gupta *et al.* 2017). Recently it was demonstrated the existence of multiple cross-talks between azole resistance prompted by CgPdr1 and ergosterol biosynthesis controlled by CgUpc2A (Vu *et al.* 2019, 2021; Vu and Moye-Rowley 2022). Interestingly, 18 documented direct targets of CgUpc2A were detected in our dataset of genes up-regulated in fluconazole-challenged ISTB218 cells (see Supplementary Table 9) including the drug-efflux pumps *CgTPO1-1* and *CgQDR2*; but also the sphingolipid flippase *CgRSB1*; *CgADH1*, encoding a cytosolic alcohol dehydrogenase; and the sterol importers *CgAUS1* and *CgTIR3* (Nakayama *et al.* 2007). Surprisingly, no over-expression of ERG genes was found in fluconazole-challenged ISTB218 cells, also described as direct targets of CgUpc2A under fluconazole stress (Vu *et al.* 2021). Neither *CgPHD1* nor *CgUPC2B* were described to be under the regulation of CgUpc2A and thus the molecular mechanism behind their over-expression in ISTB218 cells remains unclear. With the data we have available it is not possible to disclose whether the observed up-regulation of genes of the CgUpc2A regulon detected in ISTB218 cells results from a higher activity of CgUpc2A in these cells or whether this can also involve CgUpc2B or another yet uncharacterized regulator. However, this is an aspect that has to be further addressed in the future considering the role that these genes have in enhancing fluconazole tolerance.

**Fig. 5.**
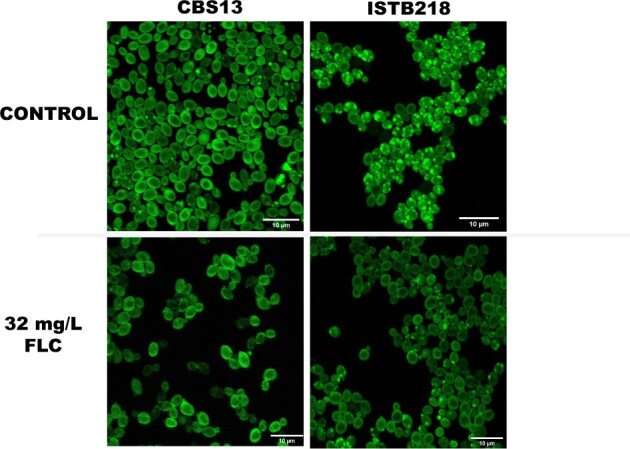
Labeling of CBS138 and ISTB218 with fillipin during growth in RPMI medium, either or not supplemented with 32 mg/L fluconazole. Cells of the 2 strains were cultivated under the same experimental setup used for the comparative transcriptomic profilings (see details in *Materials and Methods*), harvested by centrifugation, resuspended in phosphate saline buffer and incubated with filipin III for 30 min, in the dark. After this period, cells were washed with PBS, immobilized in a frame gene with agarose and imaged on a Leica TCS SP5 inverted confocal microscope. Note the differential distribution of fillipin among the strains, with a higher incidence of accumulation in internal vesicules in the azole-resistant strain ISTB218.

The results of the transcriptomic profiling of ISTB218 cells in the presence of fluconazole showed substantial differences in the expression of genes related with transport of sterols including not only the above-mentioned difference in the expression of *CgUPC2B* and of the genes of the Upc-regulon; but also in the expression of *CAGL0F05137g*, similar to the transporter of toxic sterols ScPry2; and of *CAGL0K01353g*, *CAGL0E06424g*, and *CAGL0K00715g*/*CgRTA1* genes, all encoding proteins functionally annotated as implicated in transport of sterols. In addition, a high number of SNPs were only found in the *CAGL0F05137g* allele encoded by the ISTB218 strain, this encoding a protein similar to ScLsm4 that was described to mediate the transfer of sterols between the plasma membrane and the endoplasmic reticulum (Tiwari *et al.* 2007). In agreement with these observations that suggest a differential transport of sterols in ISTB218 and in CBS138 cells, labeling with filippin, a widely used ergosterol sterol marker, reveals prominent differences (both in the presence or absence of fluconazole) with the cells of the clinical strain showing a marked distribution of the dye in what appears to be punctate internal vesicles, while in CBS138 the dye is largely distributed in the plasma membrane only being present in these internal structures in a small fraction of cells (Fig. 5). Ergosterol is synthesized in the endoplasmic reticulum and delivered to the plasma membrane using a nonvesicular mechanism that results in the equilibration of sterol pools between the two organelles. It is possible that this equilibrium between ergosterol accumulated in the plasma membrane and in the ER may be perturbed in ISTB218 cells in such a way that enhances azole tolerance. Interestingly, *Saccharomyces*  *cerevisiae* Upc2 was found to serve as a direct sensor for ergosterol becoming activated when binding of the protein to this lipid is reduced (Yang *et al.* 2015). Although the same has not been demonstrated for CgUpc2A, the high similarity between these regulators and ScUpc2, along with the recent demonstrations that perturbations in ergosterol biosynthesis, including those resulting in azole resistance, trigger activation of this regulator (Vu *et al.*  2019, 2021; Vu and Moye-Rowley 2022), rendering possible to have a similar activating mechanism in *C. glabrata*. It is thus tempting to speculate whether the observed modifications in the distribution of ergosterol in ISTB218 cells can also turn CgUpc2A (and eventually CgUpc2B) more active in these cells resulting in the over-expression of genes of the Upc-regulon in these cells.

High-throughput phenotypic analyses undertaken in *C. glabrata* have been showing that inactivation of multiple genes can result in improved azole tolerance, albeit the underlying mechanisms remain elusive (Ishchuk *et al.* 2019; Gale, Sakhawala *et al.* 2020). In this line, it was interesting to observe in our dataset of genes down-regulated in ISTB218 cells 6 fluconazole-susceptibility genes including 2 genes with a function in maintenance of mitochondrial genome (*CAGL0L12320g* and *CAGL0K05797g*), 1 predicted UDP-galactose transmembrane transporter (*CAGL0H00847g*), 1 protein phosphatase (*CAGL0F00957g*) and a functionally uncharacterized gene (*CAGL0A02299g*). Furthermore, *CAGL0J00847g*, encoding a subunit of the mitochondrial succinate dehydrogenase complex, was also found to be truncated in ISTB218, but not in CBS138. Although it is long known that loss of mitochondrial chromosome enhances azole resistance by activating CgPdr1 (Sanguinetti *et al.* 2015; Salazar *et al.*  2018, 2020), more recent results show that inactivation of specific mitochondrial functions may also result in increased azole tolerance in a CgPdr1-independent manner (Gale *et al.* 2020; Siscar-Lewin *et al.* 2021). Within this line of thinking it was also interesting the observed strong down-regulation and early truncation of the predicted aquoglyceroporines *CAGL0A01221g* and *CAGL0D00154g* since mounting evidences point to a mechanism of facilitated diffusion underlying entry of azoles to the inside of *Candida* cells (Mansfield *et al.* 2010), with the proteins/channels mediating this phenotype remaining to be identified. Interestingly, premature truncation of genes encoding aquoglyceroporines was also observed in another azole-resistant clinical strain (Vale-Silva *et al.* 2017).

The class of genes whose coding sequence differed more significantly between ISTB218 and two azole susceptible strains (CBS138 and ISTA29) were those involved in adhesion. Genes encoding adhesins were also among those suffering more dramatic changes in expression in ISTB218 and CBS138 strains, with some being potently up-regulated in the clinical strain (such as *CgAWP6* or *CAGL0H07469g*) while others are much more expressed in the laboratory strain (such as *CgEPA2* or *CgEPA6*). In most cases the differences in expression of these adhesin-encoding genes was observed both in the presence or absence of fluconazole, however, the presence of the azole exarcebated the differences (compare data in Supplementary Tables 8 and 9). Prior comparative genomic analyses involving clinical strains have shown that adhesion is under a strong selective pressure in *C. glabrata* cells in vivo (Biswas *et al.* 2017; Vale-Silva *et al.* 2017; Salazar *et al.* 2018) and although this has been attributed to the need of facilitating attachment and colonization of epithelial tissues, it is possible that such re-organization of proteins protruding from the cell envelope may positively influence resistance to azoles, for example, by restricting their entry inside the cells. In this line, it was recently described the beneficial effect of the adhesin Epa3 in azole tolerance contributing, among other aspects, to reduce the internal concentration of the azole (Cavalheiro *et al.* 2019).

**Fig. 6.**
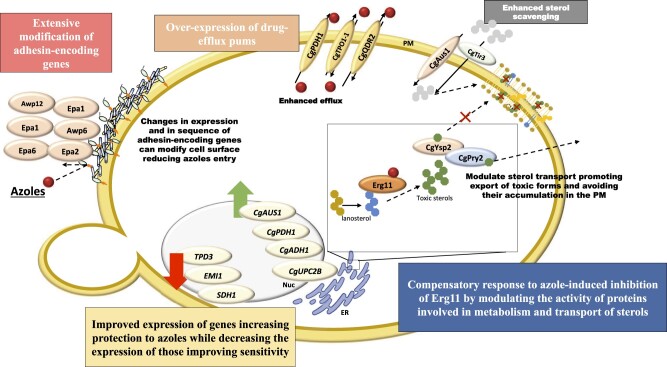
Schematic representation of the mechanisms suggested to contribute for azole resistance in the resistant isolate *C. glabrata* ISTB218, encoding a wild-type *CgPDR1* allele (that is, having SNPs that had been identified both in azole resistant and in susceptible clinical strains), as suggested by comparative transcriptomic and genomic analyses.

On overall this study contributes to improve current understanding of acquired azole resistance in *C. glabrata* clinical isolates either by identifying 2 novel gain-of-function CgPdr1 variants that were not previously demonstrated, I803T and I392M, and by disclosing insights into possible CgPdr1-independent responses. Collectively, the data from the OMICS profiling performed on ISTB218 cells, in the presence or absence of fluconazole, suggest that the azole-resistance phenotype can result from a multitude of factors that are summarized in Fig. 6. These factors include the over-expression of the azole-protection genes *CgAUS1, CgADH1*, the drug efflux pumps CgPhd1, CgQdr2 and CgTpo1p-1; and *CAGL0L03377g, CAGL0L07480g, CAGL0G08624g*, *CAGL0K01991g*, and *CAGL0J01661g*, whose function in azole tolerance remains unknown. An alteration in the distribution of ergosterol between the ER and the plasma membrane in ISTB218 and in CBS138 cells, suggested by a different labeling with fillipin and by extensive modification in the expression and sequence of various genes encoding proteins involved in sterol transport, is also hypothesized to influence the lower susceptibility to fluconazole of ISTB218 cells (and eventually affect activity of CgUpc2A and/or CgUpc2B). This response can facilitate the export and/or the compartmentalization of toxic sterols thus avoiding the toxic effects prompted by their accumulation in the plasma membrane of ISTB218 cells. Finally, the extensive modification of the cellular envelope prompted by the modification in the expression and in amino acid sequence of adhesins, along with the inactivation of azole-susceptibility genes, are other mechanisms suggested by our OMICS analyses that are likely to underlie azole tolerance of ISTB218 cells. The next step that needs to be taken is a subsequent detailed genetic analysis to understand how these different mechanisms contribute, alone or in combination, for the azole-tolerance phenotype of the ISTB218 strain; however, such thorough analysis is only possible upon the identification of the more promising candidates, an information that is uncovered for the first time in this work and that is expected to pave the way for a deeper understanding of the acquisition to azoles in vivo in *C. glabrata*.

## Data availability

Strains and plasmids described in Supplementary Table 1 are available upon request. Both the transcriptomic and genomic data were deposited in public access databases, namely GEO (accession number GSE166841) and NCBI (Bioproject PRJNA699880).

Supplemental material is available at *G3* online.

## Supplementary Material

jkac110_Supplementary_Figure_S1

jkac110_Supplementary_Figure_S2

jkac110_Supplementary_Figure_S3

jkac110_Supplementary_Material
