# Supplementary material for: Disclosing azole resistance mechanisms in resistant Candida glabrata strains encoding wild-type or gain-of-function CgPDR1 alleles through comparative genomics and transcriptomics
Source: G3 (Bethesda). 2022 May 9;12(7):jkac110. doi: 10.1093/g3journal/jkac110 (PMC9258547; doi:10.1093/g3journal/jkac110)
Supplement: jkac110_Supplementary_Figure_S1 [file jkac110_supplementary_figure_s1.pdf]

A

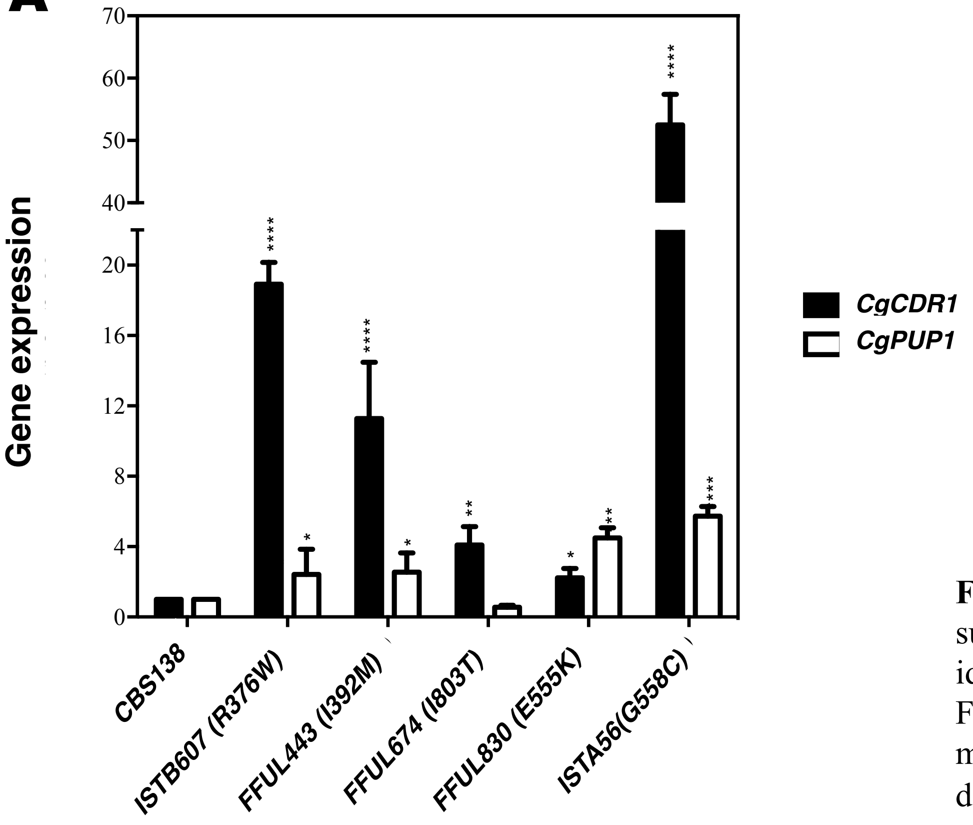

Figure S1. Expression of *CgCDR1* and of *CgPUP1* in the azole-susceptible strain CBS138 and in the azole-resistant strains identified in our phenotypic screening, including FFUL443 and FFUL674. In this experiment the cells were cultivated in RPMI medium, harvested in mid-exponential phase and then used for downstream analysis as detailed in materials and methods. The level of expression registered in the CBS138 was set at 1 and the other values compared with that. The transcript levels of *CgRDN5* were used as internal control
