# Supplementary material for: Disclosing azole resistance mechanisms in resistant Candida glabrata strains encoding wild-type or gain-of-function CgPDR1 alleles through comparative genomics and transcriptomics
Source: G3 (Bethesda). 2022 May 9;12(7):jkac110. doi: 10.1093/g3journal/jkac110 (PMC9258547; doi:10.1093/g3journal/jkac110)
Supplement: jkac110_Supplementary_Figure_S2 [file jkac110_supplementary_figure_s2.pdf]

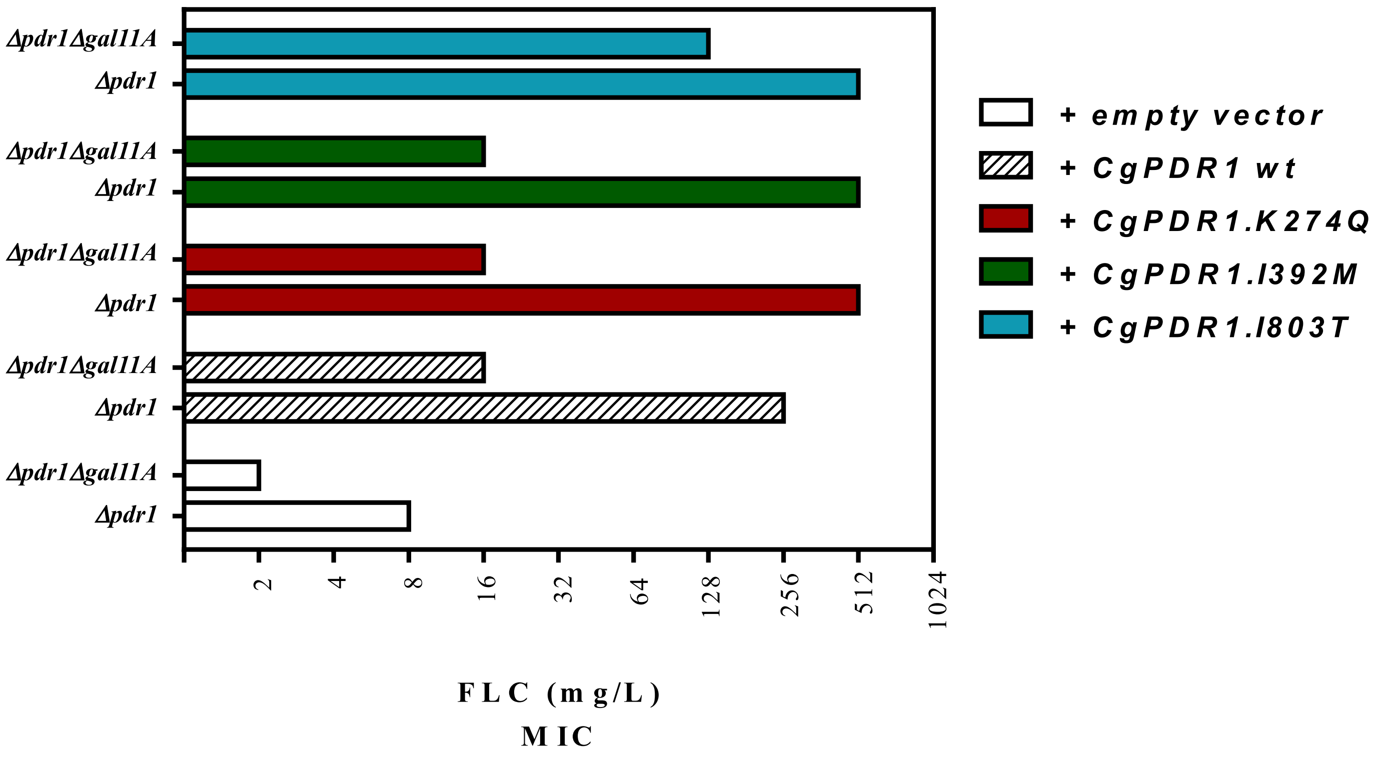

Supplementary figure S2. MIC for fluconazole in *C. glabrata* Δ*pdr1* or Δ*pdr1*Δ*gal11A* cells expressing different versions of the *CgPDR1* allele.
