# Supplementary material for: Disclosing azole resistance mechanisms in resistant Candida glabrata strains encoding wild-type or gain-of-function CgPDR1 alleles through comparative genomics and transcriptomics
Source: G3 (Bethesda). 2022 May 9;12(7):jkac110. doi: 10.1093/g3journal/jkac110 (PMC9258547; doi:10.1093/g3journal/jkac110)
Supplement: jkac110_Supplementary_Figure_S3 [file jkac110_supplementary_figure_s3.pdf]

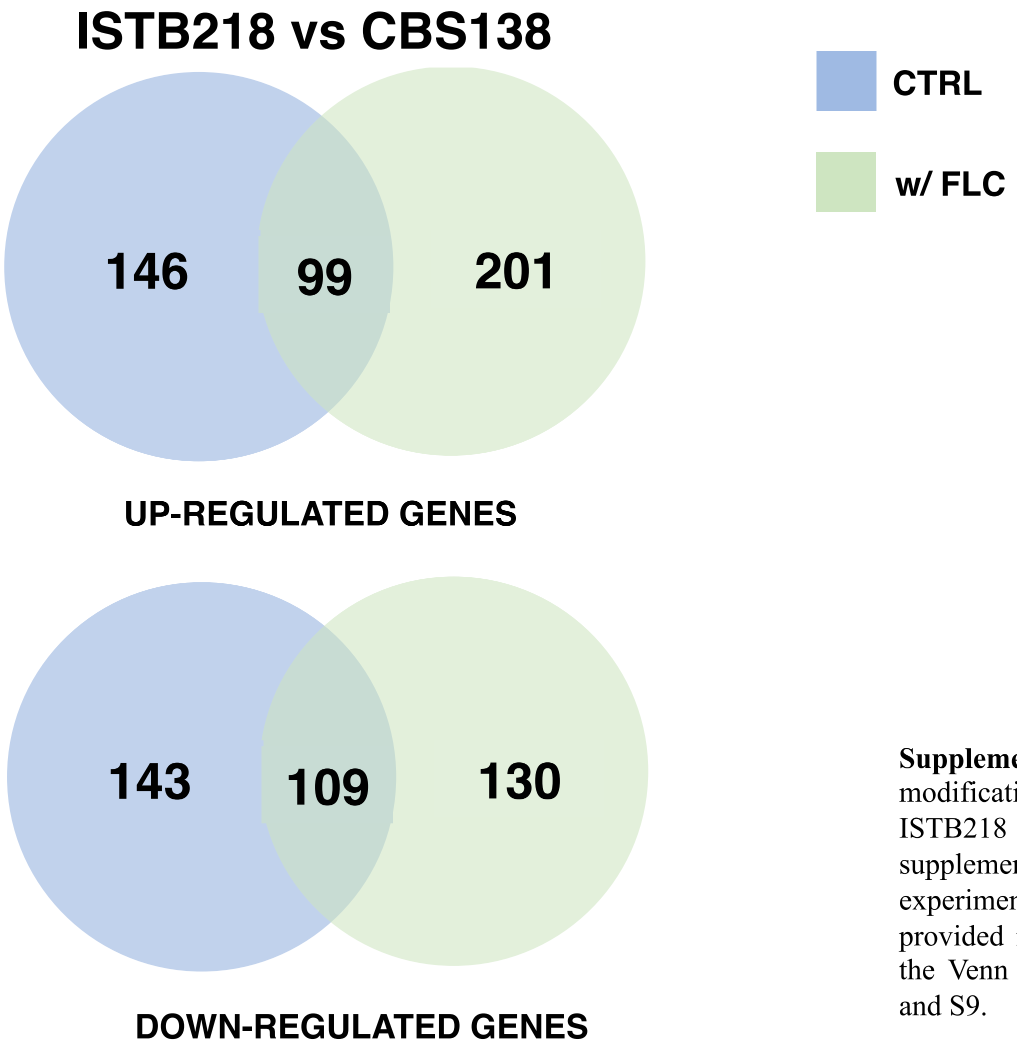

Supplementary figure S3. Venn diagram describing the modifications occurring in the transcriptome of CBS138 and ISTB218 cells cultivated in RPMI medium either or not supplemented with 32 mg/L fluconazole. Details on the experimental setup used to grow and harvest the cells are provided in materials and methods. The data used to build the Venn diagram is available in supplementary tables S8 and S9.
